# Dual Diagnoses and Health Biomarkers: Diabetes and ADRD Among Hispanic Older Adults on the US-Mexico Border

**DOI:** 10.1093/geroni/igaf122.263

**Published:** 2025-12-31

**Authors:** Hyeran Chung, Alfonso Rojas-Alvarez, Brian Downer

**Affiliations:** The University of Texas at El Paso, El Paso, Texas, United States; The University of Texas at El Paso, El Paso, Texas, United States; UTMB, Galveston, Texas, United States

## Abstract

Hispanic older adults near the US-Mexico border face significant healthcare challenges, especially in managing chronic conditions like diabetes and Alzheimer’s Disease or Related Dementias (ADRD). Having both conditions complicates disease management and increases the likelihood of poor outcomes. However, little is known about how dual diagnoses affect health biomarkers, particularly in racially and ethnically marginalized communities along the border. This study explores the associations between dual diagnoses and key biomarkers related to diabetes and ADRD to better understand the health disparities faced by this population. Using the Hispanic Established Populations for the Epidemiologic Study of the Elderly (HEPESE) and Medicare administrative data (n = 718), we examine the relationship between dual diagnoses and biomarkers such as HbA1c and cognitive outcomes. Descriptive and regression analyses compare differences in these biomarkers among those with dual diagnoses and those with a single diagnosis. Proximity to the border is considered a moderating variable based on our previous findings about caregiver and community resources impacting health outcomes. Preliminary results show that Hispanic older adults with both diabetes and ADRD near the US-Mexico border have worse biomarker profiles, including poor glycemic control (elevated HbA1c) and more severe cognitive impairment. These disparities will be more pronounced near the border, suggesting that healthcare access and structural barriers may worsen disease progression. This project highlights the compounded health challenges of Hispanic older adults with dual diagnoses in the US-Mexico border region. It provides insights into healthcare disparities that can inform future policies to improve health outcomes.

